# Diabetes Mellitus in the Transplanted Kidney

**DOI:** 10.3389/fendo.2014.00141

**Published:** 2014-08-27

**Authors:** Vasil Peev, Jochen Reiser, Nada Alachkar

**Affiliations:** ^1^Department of Medicine, Rush University School of Medicine, Chicago, IL, USA; ^2^Department of Medicine, The Johns Hopkins University School of Medicine, Baltimore, MD, USA

**Keywords:** diabetes mellitus, diabetic nephropathy, kidney-transplant, podocyte, new onset of diabetes after transplant, suPAR, mTORC1 signaling, B17 receptor

## Abstract

Diabetes mellitus (DM) is the most common cause of chronic kidney disease and end stage renal disease. New onset diabetes mellitus after transplant (NODAT) has been described in approximately 30% of non-diabetic kidney-transplant recipients many years post transplantation. DM in patients with kidney transplantation constitutes a major comorbidity, and has significant impact on the patients and allografts’ outcome. In addition to the major comorbidity and mortality that result from cardiovascular and other DM complications, long standing DM after kidney-transplant has significant pathological injury to the allograft, which results in lowering the allografts and the patients’ survivals. In spite of the cumulative body of data on diabetic nephropathy (DN) in the native kidney, there has been very limited data on the DN in the transplanted kidney. In this review, we will shed the light on the risk factors that lead to the development of NODAT. We will also describe the impact of DM on the transplanted kidney, and the outcome of kidney-transplant recipients with NODAT. Additionally, we will present the most acceptable data on management of NODAT.

## Introduction

As the epidemic of diabetes mellitus (DM) is growing, diabetic nephropathy (DN) remains the leading cause of end stage renal disease (ESRD) in the American population [US Renal Data System (USRDS) data 2012]. DN and ESRD as its consequence, poses a major healthcare problem especially in developed countries, and as such it represents a great financial burden to the society. In the United States, over 21 million people, or 7% of the general population, are estimated to have diabetes. To date, despite aggressive research conducted in the early diagnosis and treatment of DN, the vast majority of diabetic patients eventually progress to ESRD. For these patients, either dialysis or renal transplantation remain the only options of survival.

Renal transplantation has been considered the therapy of choice in suitable ESRD or pre-ESRD patients since it confers a better survival benefit to these patients compared to dialysis (1–3). Despite renal transplantation addressing the problem of imminent renal failure, DM and DN remain prevalent among kidney-transplant patients leading in some cases to allograft loss and contributing to overall patient’s mortality. DM may represent a sequela of the preexisting condition leading to ESRD or can develop as *de novo* after kidney or any other solid organ transplantation in transplant recipients.

Diabetic nephropathy was the etiology of ESRD in approximately 23% of kidney-transplant recipients in the United States in 2008 (4). These numbers unfortunately continue to grow as the number of diabetic patients in the general population increases.

## New Onset of Diabetes Mellitus after Kidney-Transplant

The incidence of new onset diabetes mellitus after transplant (NODAT) is variable, ranging between 10 and 46% depending on the study design and the definition of NODAT (5–7). More specifically, NODAT has been reported to occur in 4–25% of renal transplant recipients, 2.5–25% of liver transplant recipients, 4–40% of heart transplant recipients, and 30–35% of lung transplant recipients (8–11). In order to establish more precise diagnosis of NODAT, international consensus guidelines defined the criteria for NODAT and these include the following: symptoms of diabetes and random plasma glucose ≥200 mg/dL (11.1 mmol/L), fasting plasma glucose ≥126 mg/dL (7.0 mmol/L), and 2-h plasma glucose ≥200 mg/dL (11.1 mmol/L) during an oral glucose tolerance test (8). If at any time point either of these criteria in post transplant patient is met, the diagnosis of NODAT can be established. Levels of glycosylated hemoglobin A1c are unreliable marker of NODAT during the first three to six post transplant months, given the fact that many chronic kidney disease (CKD) stage 5 and ESRD patients are anemic at baseline when receiving a kidney-transplant. Many patients undergo renal and other solid organ transplantation, hence only a subgroup of patients will develop NODAT. It remains poorly understood what predisposes this subgroup of patients to the development of NODAT. The literature describes various modifiable and non-modifiable risk factors for development of NODAT. Non-modifiable risk factors include age, race, genetic background, family history of diabetes, and previous glucose intolerance. Modifiable risk factors are obesity, hepatitis C virus (HCV), cytomegalovirus infections, and immunosuppressive drugs (12, 13). The risk of NODAT development increases with time from transplantation. Therefore, early detection and prompt action are essential in reducing the risk of NODAT and its complications (14).

Among the non-modifiable risk factors age is considered the strongest risk factor for development of NODAT (6, 12, 15). A study by Cosio et al. (7) that included 2078 allograft recipients, showed that individuals older than 45 were 2.9 times more likely to develop NODAT. Data from the USRDS showed that first kidney-transplant recipients with ages between 45 and 59 years had a relative risk (RR) for NODAT of 1.9 (95% confidence interval (CI) 1.73–2.09; *P* < 0.0001), whereas, individuals ≥60 years had a risk of 2.6 (95% CI 2.32–2.92; *P* < 0.0001) (12, 15). Age increased the risk for development of diabetes 1.5-fold for every 10-year increase in age (12, 16).

## Risk Factors of NODAT

Obesity represents a modifiable risk factor and has consistently been shown to be strongly associated with development of NODAT (6, 15, 17). Data from the USRDS revealed that obese patients (BMI ≥30 kg/m^2^) have an RR for NODAT of 1.73 (95% CI 1.57–1.90; *P* < 0.0001), this being, along with age, one of the most consistent risk factors (6, 12). The molecular mechanisms of obesity have been studied in detail in the non-transplant population where chronic inflammatory markers synthesis is upregulated. Adipose tissue is known to produce adipocytokines, including leptin, tumor necrosis factor alpha (TNF-α), interleukins, and adiponectin (18, 19). Activation of the TNF-α system has been associated with insulin resistance through the generation of defects in the phosphorylation of the receptor and decreasing the expression of insulin-sensitive glucose transporters. Induction of IL-6 synthesis has been associated with alterations in glucose tolerance and is possibly a predictor of DM2 (19, 20). Adiponectin is a 30-kDa, collagen-like protein synthesized by adipocytes. High adiponectin concentrations have been associated with an independent reduction in the risk of developing type 2 DM in a healthy population (19, 21). In transplant recipients, a low pre-transplant serum concentration of adiponectin was described as an independent risk factor for the development of NODAT (22). A recent study found that for every 1 μg/mL decrease in adiponectin concentration, the risk of developing NODAT is increased by 13% (19).

Race also plays an important role with respect to NODAT development. Even though most of the transplant centers through out the world expose their patients to similar immunosuppressive induction and maintenance protocols, African American and Hispanic patients appear to have a significantly higher risk for development of NODAT compared to Caucasians. Likely, genetic polymorphisms among Black and Hispanic transplant recipients allow for more common disease prevalence compared to their Caucasian counterparts. These polymorphisms remain a subject of research, however precise description is lacking in the transplant literature.

Additionally, infections are also described as culprits of NODAT. Transplanted patients at baseline are more susceptible to infections than the general population. A chronic HCV infection (either acquired peri-operatively from the donor or present in the recipient at the time of transplantation), especially in a setting of uncontrolled viremia, poses a risk factor for NODAT development. The USRDS registry confirmed that the 1-year incidence of NODAT in HCV-positive patients at transplantation was significantly higher compared to the HCV-negative patients (25.6 vs. 15.4%; *P* < 0.0001) (6), clearly demonstrating higher risk for NODAT development in HCV carriers. Recent basic science studies have demonstrated that the HCV elicits an apoptosis-like death of pancreatic beta-cells through endoplasmatic reticulum stress-involved, Caspase 3-Dependent Pathway (23). Interferon has been the drug of choice for treatment of hepatitis C infection in the non-transplant population for several decades. Unfortunately, its use in the HCV infected transplant patients has been widely avoided due to its propensity to elicit acute rejection in the allograft. The novel drugs (protease and nucleotide analog inhibitors) recently released on the market for treatment of HCV infection in non-transplanted patients are still lacking official FDA approval in the transplant cohort. Their use in this cohort has been strictly off label and transplant center dependent. Thus, their prime time for this clinical indication is likely to come in the near future. It would be interesting to see if this will have a positive impact on decreasing the incidence of NODAT in the transplant recipients.

## Immunosuppression Drugs

In addition to the above mentioned factors influencing NODAT development, several immunosuppressive agents that are commonly used in the transplant arena have been noted to have diabetogenic potential. Herein, commonly used drugs include glucocorticoids, calcineurin inhibitors (CNIs) including tacrolimus (TAC) and cyclosporine (CYC), as well as the mammalian target of rapamycin (mTOR) inhibitors (sirolimus and everolimus). The diabetogenic effect of all of these drugs has been explored in detail and their effect on development of NODAT has been elucidated in several major studies.

Glucocorticoids, as the oldest immunosuppressive agents, have a strongest diabetogenic potential, which is dose dependent. The predominant factor for causing PTDM by corticosteroids seems to be the aggravation of insulin resistance, however, several studies have displayed deleterious effects on insulin secretion and beta-cells apoptosis (24). The precise mechanisms of glucocorticoid-induced insulin resistance are not well understood. *In vivo* and *in vitro* animal studies demonstrate initiation of glucocorticoid related insulin signaling cascade in skeletal muscles, resulting in reduced glucose uptake and glycogen synthesis (25, 26). *In vitro*, the effect of glucocorticoids on β-cell lines has been studied in detail. Glucocorticoids were shown to reduce the expression of GLUT 2 and glucokinase, thereby impairing glucose-stimulated insulin secretion (24, 26). Further, Dexamethasone was shown to stimulate transcription of serum and glucocorticoid-inducible kinase 1, upregulating the activity of voltage-gated K^+^ channels and leading to reduced Ca^2+^ entry through voltage-gated Ca^2+^ channels with resultant decreased insulin release (26, 27). In isolated rat islets, dexamethasone decreases the activation of protein kinase C through inhibition of the diacylglycerol–phospholipase C pathway (26, 28).

Additionally, there is some conflicting data regarding the benefit of early corticosteroid withdrawal vs. steroid continuation protocols with respect to NODAT manifestation.

A large randomized controlled study found that early steroid withdrawal does not confer any significant advantage compared to steroid continuation, with the remark that fewer patients with early steroid withdrawal required insulin for NODAT at 5 years [4/107 (3.7%) vs. 10/86 (11.6%), *P* = 0.049] (29). On the other hand, large retrospective study involving more than 25,000 transplant recipients reported significant benefits of early steroid withdrawal when compared to a steroid-containing regimen with respect to NODAT. The cumulative incidence of NODAT within 3 years post-transplant was 12.3% in steroid-free vs. 17.7% in steroid-containing regimens, *P* < 0.001. Overall, steroid-containing regimens carried a 42% increased risk for NODAT at the time of hospital discharge (30).

Calcineurin inhibitors are also commonly prescribed drugs in the transplant arena. Both CYC and TAC administration correlates strongly with NODAT development, however, TAC appears to have more pronounced diabetogenic effect as demonstrated in prospective and retrospective studies (31, 32). This was observed in kidney, heart, liver, and lung transplants (24). Interestingly, some of the basic science studies involving these agents are not fully supportive of these clinical findings (33).

Calcineurin inhibitors induce NODAT by variety of mechanisms, including decreased insulin secretion and a direct toxic effect on the pancreatic beta-cells. The effect on beta-cells survival implicates the direct effect of CNIs on the nuclear factor of activated T-cell (NFAT) signaling. CNIs regulate the dephosphorylation of (NFAT) protein and CREB (cAMP-responsive element-binding transcription factor) activity-2 (TORC2). The dephosphorylation of these proteins regulates several target genes [insulin, Glut2, (pancreatic and duodenal homeobox 1 (Pdx-1), insulin receptor substrate-2 (Irs2), cyclin D1, cyclin D2, cyclin-dependent kinase 4 (CDK4), etc.], which are critical in β-cell survival, replication, and function. TAC binds intracellularly to FK506-binding protein 1B (FKBP1B) before docking in the calcineurin binding site (Cnb1) of calcineurin, thus inhibiting calcineurin and its downstream pathways and decreasing β-cell replication and survival (26). Moreover, CYC induces inhibition of calcineurin activated leucine zipper-bearing-kinase, leading to beta cell apoptosis (34). Further, mitochondria play a key role in the insulin secretion mainly by providing ATP supply. CYC binds readily to cyclophilin D in the mitochondrial permeability transition pore and blocks the opening of this channel on the mitochondrion and thereby reduces the cytoplasmic free-Ca^2+^ concentration thus interfering with glucose-stimulated insulin secretion (26, 35).

Finally, even though with less frequency, mTOR inhibitors continue to be used among transplant patients despite clinical evidence of their use being associated with greater risks of allograft failure and recipient death compared with a CNI-based regimen (36). Additionally, mTOR inhibitors have been associated with significant risk for NODAT development, especially in combination therapy with TAC, thus with a tendency of sirolimus contributing more to the NODAT development rather than TAC (37).

The effects of sirolimus on the function and survival of β-cells appears paradoxical based on animal studies and vitro studies with cell lines or human islets. *In vitro* sirolimus is noted to increase the insulin content in human islet cells (38) as well the secretion in both basal (50%) and stimulated (40%) states in mini pigs *in vivo* (39). On the other hand, additional *in vitro* studies have shown that sirolimus may facilitate the opening of ATP sensitive potassium channels thereby impairing the insulin secretion (40) in addition to suppressing the glucose-stimulated insulin secretion via direct inhibition of Krebs cycle and decrease of mitochondrial ATP production (41). Further, there is convincing evidence that sirolimus may disrupt regeneration and proliferation of islets, most likely via direct inhibition of the mTOR complex 1 (mTOR C1) signaling and its downstream regulatory effect on cyclin-dependent kinase 4, ultimately leading to reduced cyclin D2 and D3, which are critical regulators of β-cell cycle, proliferation, and mass (38, 42). In summary, the effects of sirolimus on insulin secretion remain the subject of further investigation.

There are no data so far to indicate that mycophenolate and azathioprine are involved in the development of NODAT.

## Diabetic Nephropathy after Kidney-Transplant

Diabetic nephropathy occurs in the transplanted kidney after approximately 5.9 years (43), in patients with pre-transplant DM and those who develop NODAT. In a study of 58 kidney-transplant recipients, 74.1% had history of DM before kidney transplantation and 25.9% had NODAT, of those whom DN histologic findings developed, 69.6% were in patients with history of DM and 30.4% were in NODAT patients. The time from transplantation to the development of DN was slightly longer in NODAT patients (6.68 ± 3.86 vs. 9.93 ± 3.07 years, *P* = 0.05); however, as expected, the duration of diabetes was similar in the two groups at the time of histologic findings of DN (6.68 ± 3.86 vs. 5.90 ± 3.13 years, *P* = 0.66) (43).

The pathological findings of DN post kidney transplantation are in most part similar to those of typical DN in native kidneys. Thickening of GBM and the tubular basement membrane constitutes the first sign of DN. Mesangial matrix expansion develops later on. The extracellular matrix accumulates over time and forms nodular mesangial changes that gradually lead to the compression of the associated glomerular capillaries resulting in glomerular sclerosis and obliteration of capillary lumina. Hyalinosis in the afferent and efferent arteriolar occurs simultaneously with the glomerular lesions leading also to tubulointerstitial chronic changes (44). DN in the transplanted kidney, however, frequently associate with vascular and tubulointerstitial changes caused by allograft rejection, viral infection, or CNI nephrotoxicity, which differentiates it from the DN in the native kidney.

In spite of the extensive data on the pathways that lead to DN in the native kidney, there is still a paucity of such data on DN after kidney transplantation. Although, we believe that the same mechanisms lead to DN in native kidney contribute to the development of post transplant DN, there have been no studies confirming these mechanisms in the transplanted kidney. However, there are few pathways that have been described in the initiating and developing of DN; these may have some significance in the transplanted kidney. Herein, will discuss two of these pathways.

Recently, there have been major changes in our understanding of DN development in the native kidney, with a major focus on the podocyte as the initial site of injury (45), which leads later to the progression of the classic changes of diabetic nodular glomerular sclerosis and interstitial fibrosis.

In a recent study by Fiorina et al., the investigators described the role of podocyte B7-1 in the podocyte injury that results from hyperglycemia (46). The authors found that B7-1 upregulation was induced by hyperglycemia; this upregulation was found to be mediated by activation of the 110-kDa catalytic PI3Kα subunit. Furthermore, the addition of CTLA4-Ig such as abatacept prevented cytoskeleton disruption and adhesion in podocytes that were exposed to hyperglycemia *in vitro* (46). This data can be of a significant importance in kidney transplantation field. Belatacept, a newer CTLA4-Ig with higher affinity to B7-1 has been approved recently as a maintenance immunosuppressive therapy in kidney-transplant. Hence, it will be of a great interest to evaluate the effect of belatacept in preventing the developing of DN after kidney transplantation.

A second pathway that has been described as a possible contributor to the development and progression of DN in the native kidney is the mTOR. Recent studies suggested that the mTOR pathway of the podocytes plays an important role in the underlying mechanisms of the progression of glomerular diseases (47) and DN (48). In a recent data by Gödel et al., the investigators confirmed that mTOR complex (mTORC) 1 and 2 have crucial roles in the podocyte function. Deletion of mouse podocytes mTORC 1 and 2 induced significant proteinuria and lead to the progression of glomerulosclerosis (48). On the other hand, patients with DN have a significant activation of the podocytes mTOR that associated with early glomerular hypertrophy and hyperfiltration (48). Therefore, there is a potential utilization of mTOR inhibitors such as rapamycin in the prevention of developing DN. Rapamycin has been in use for many years as a maintenance immunosuppressant to prevent kidney-transplant rejection.

A third pathway implies the role of circulating factors that are known to be involved in causing proteinuria. For example, circulating soluble urokinase plasminogen activator receptor uPAR (suPAR) that is known as one of the culprits in native and recurrent Focal and Segmental Glomerulosclerosis (FSGS) has recently been shown to play an active role in patients with DN (49). There, increased suPAR serum levels cause podocyte apoptosis through its association with acid sphingomyelinase-like phosphodiesterase 3b (SMPDL3b) on podocytes. Additional clinical studies further support this concept showing that suPAR is a predictor of proteinuria in patients with DM (50). The neutralization of suPAR may thus be a novel approach to treat DN in the native and possibly also in the transplanted kidney.

## Management of NODAT

The management of NODAT requires a multifaceted approach given that this condition affects multiple organs other than the allograft itself. Other than extensive counseling of pre-transplant patients regarding the higher odds of developing NODAT (especially in high risk groups, as outlined above) and recommendation for implementation of general pre-transplant measures (like weight loss, physical activity, and dietitian referral), most of the transplant centers in the United States have abandoned the screening of transplant recipients with oral glucose tolerance test as this would possibly affect the candidacy for transplantation. Post transplantation screening for NODAT is, however, recommended for all solid organ recipients. Namely, most of the transplant centers would screen the recipients after transplant with weekly fasting glucose testing in the first month after transplantation and continue screening at 5, 6, and 12 months. After this period even though the risk of NODAT is somewhat lower, at least yearly testing with either glycosylated hemoglobin or fasting glucose is recommended.

Early diabetes specialist referral, adequate glycemia control as well as treatment of comorbid conditions remain the backbone of medical approach to this condition. Further, good control of glycemia may even decrease the risk for rejection (51). In patients, who develop DN with overt micro and macroalbuminuria, strict glycemia control in addition to the use of angiotensin inhibitors and statins remains strongly recommended. In transplant patients other than the aforementioned measures extrapolated from the general population, the benefit of decreasing immunosuppression with respect to NODAT prevention and treatment should be carefully weighted against the risk of provoking a rejection in the allograft. Switching from one class of immunosuppressive medication to other should be individualized since the more diabetogenic transplant medications may have other advantages to the longevity of the allograft compared to their competitors.

## Outcome of Kidney-Transplant Recipients with NODAT

Even though the two main causes of kidney-transplant loss are chronic allograft nephropathy and death with a functioning allograft, NODAT is strongly associated with impaired patient survival (6, 52) as well as increased cardiovascular mortality (52, 53). Studies demonstrate that kidney-transplant recipients with NODAT are at a two- to three-fold increased risk of fatal and non-fatal cardiovascular disease events as compared with non-diabetic patients (53, 54). In addition, large retrospective registries have concluded that NODAT is a strong, independent predictor of global mortality, graft failure, and death-censored graft failure (6).

The mechanism by which NODAT influences the allograft survival is not well understood, other than predisposing patients to recurrent infections and acute rejection especially when attempts are made to decrease immunosuppression in order to minimize the diabetogenic effect of medications like TAC. In addition to this, NODAT likely contributes to recurrence of DN in the allograft. Similar to the general population, diabetic complications are also commonly encountered in patients with NODAT including ketoacidosis, neurologic, and ophthalmic complications as well as recurrent hypoglycemia and shock (55).

## Summary

In summary, NODAT is a common and serious condition that affects the overall health and the survival of a transplant recipient. Inability to control NODAT is associated with significant allograft failure as well as overall transplant recipient morbidity. Strenuous efforts must be undertaken to minimize its detrimental effect on the comorbid conditions that develop as a consequence of NODAT. We are looking forward to newer transplant protocols and medications that do not have the diabetogenic side effects of the ones currently used among the transplant patients and many such are currently underway.

## Conflict of Interest Statement

Jochen Reiser is an inventor on pending and issued patents related to the modification of suPAR. He stands to gain royalties from their commercialization. The rest of the authors have no conflict of interest.
